# Communicating with Adolescents and Young Adults about Cancer-Associated Weight Loss

**DOI:** 10.1007/s11912-019-0765-7

**Published:** 2019-02-04

**Authors:** Joanne Reid, Clare McKeaveney, Peter Martin

**Affiliations:** 1 School of Nursing and Midwifery, Queen’s University Belfast, Medical Biology Centre, 97 Lisburn Road, Belfast, BT9 7BL UK; 2 School of Medicine, Geelong Waurn Ponds Campus, Locked Bag 20000, Geelong, VIC 3220 Australia

**Keywords:** Adolescents and young adults, Cancer-associated weight loss, Cachexia

## Abstract

**Purpose of Review:**

Over the past two decades, advances have been made in understanding the pathophysiology of cancer-associated weight loss, termed “cachexia.” To date, there is no proven effective intervention to completely reverse cachexia and there are no approved drug therapies to treat it. This paper will review relevant literature in relation to communicating with adolescents and young adults about cancer-associated weight loss.

**Recent Findings:**

Adolescents and young adults (AYAs) who have cancer are a unique group of patients due to their stage of development and maturity.

**Summary:**

This article outlines issues specific to this patient cohort that need to be considered to better understand the impact of cachexia and explore pertinent matters when communicating with AYAs in relation to cachexia.

## Introduction

Cancer-associated weight loss or cachexia is a “multifactorial syndrome characterized by an ongoing loss of skeletal muscle mass, with or without loss of fat mass that cannot be fully reversed by conventional nutritional support and leads to progressive functional impairment” ( [1], p.489). The pathophysiology of cancer cachexia is multifarious and reflective of this a multimodal approach to its management has been advocated [2]. However, to date, a successful treatment regime has not been definitely tested and there is a dearth of local practice guidelines and approved treatments for cancer cachexia. Previous research has uncovered the holistic impact of cancer cachexia for both patients and their carers; however, the vast majority of this work has been conducted with adult cancer patients. For adolescents and young adults (AYAs), the impact of cancer cachexia is likely to be distinct from older age groups given the challenges of this development stage. How we communicate with this group in relation to cachexia is particularly relevant as AYAs are regarded as particularly vulnerable [3]. Adolescence and early adulthood is known to be a distinct and complex development stage in a person’s life not only because of cognitive, emotional, and physical changes but also because of psychosocial challenges related to self-identity, relationships with family and peers, development of autonomy, sexuality, and education/work-related issues [4].

## Defining Adolescents and Young Adults

The term “adolescents and young adults” has been variously described in the literature with differing age ranges. For example, previous cancer research has operationally defined AYAs as follows: 10–19 [5] 10–26 [6]; 11–21 [7]; 11–24 [8]; 13–21 [9]; 13 plus [10]; 14–39 [11, 12]; 15–25 [13, 14]; 15–29 [15]; 15–30 [16]; 15–34 [17]; 15–39 [18–25]; 15–29 [26]; 16–29 [27, 28]; 16–30 [29, 30]; 18–25 [31]; 18–35 [32]; 18–39 [33]; 18–40 [34]; 18–44 [35]; 18–45 [36].

No formal unified agreement of the age rage for AYAs exists within the literature reviewed. As a result, the aforementioned age ranges are used interchangeably and this makes comparisons across research studies all the more complex. However, what is clear is that this stage of life marks key transition years from adolescent to adulthood and for most persons are the most dynamic and confounding years of life [4]. It is recognized that AYAs with cancer are a unique cohort due to their distinct biology, maturing hormonal and personal development, transitions in autonomy, demands in education and the workplace, and their place within the family/family responsibilities [37].

## Cancer Incidence and Research in Adolescents and Young Adults

Globally, cancer remains a major cause of both morbidity and mortality across all age groups. Given there are more than one million new diagnoses of cancer worldwide in AYAs per annum [38], the global burden is greater than in all other age groups. Cancer is generally a disease associated with increasing age and thus the majority of research has focused on such populations. As such, research into the impact of cancers that occur at the juncture between pediatric and adult oncology remains in its infancy [39]. It is widely accepted that AYAs with cancer should have age-appropriate care to meet their needs and such care should be evidence based [40]. However, research involving AYAs is seldom the focus of cancer studies and this population is known to be under-researched [37, 41]. This is in stark contrast to the fact that the number of life-years affected by cancer is greater for AYAs than in any other age group [42]. It is also worth noting that internationally, recruitment of AYAs above the age of 15 into cancer trials is poor. Most notable, this is seen to directly relate with deficits in survival gains for patients aged 15–39 years [43]. Thus, while acknowledging the needs for additional research with this cohort, any such research needs to focus on innovative ways of engaging AYAs by better understanding the barriers and facilitators to recruitment and retention to maximize scientific advances and understanding of cancer-associated weight loss in this population.

## Cancer-Associated Weight Loss

**Fig. 1:**
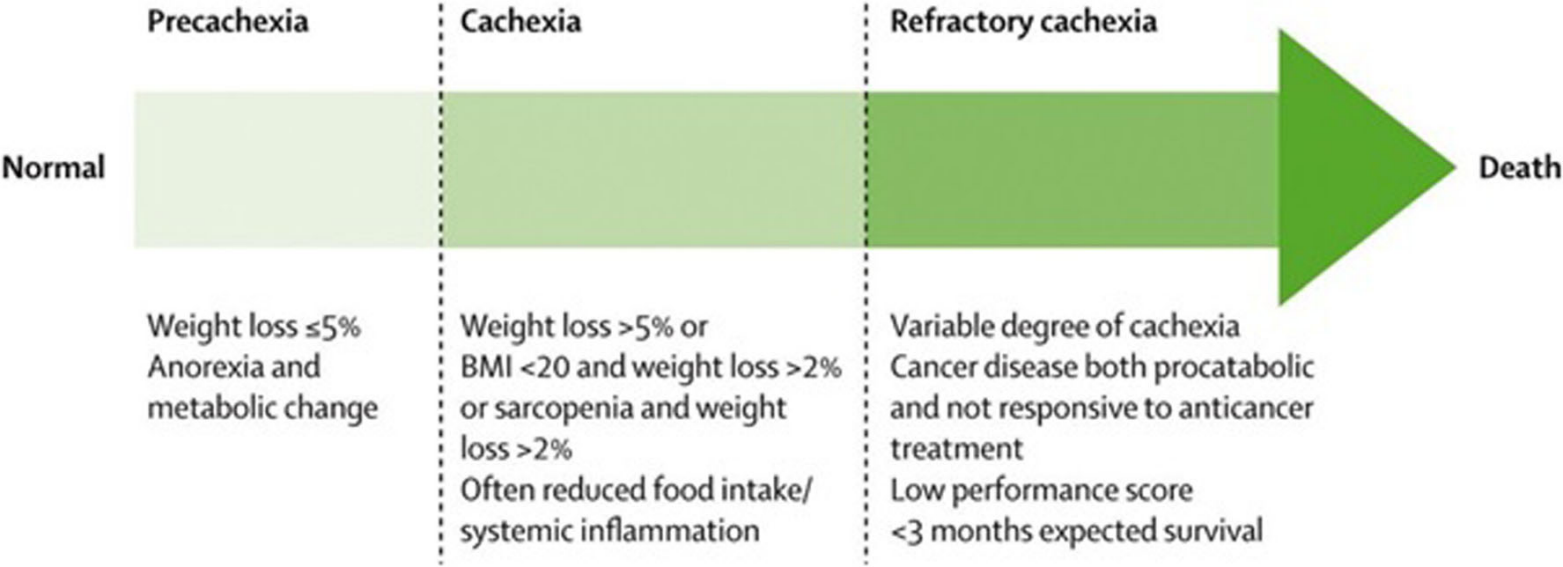
Stages of cachexia. Reprinted from *The Lancet Oncology*, Volume 12, Fearon K, et al., “Definition and classification of cancer cachexia: an international consensus,” pages 489–95, ©2011, with permission from Elsevier

Patients with cancer are known to experience weight loss for a variety of reasons including, stress/anxiety, poor symptom management, and treatment-related factors. However, cancer-associated weight loss or cachexia is multifactorial syndrome characterized by progressive and involuntary weight loss and loss of muscle mass. There are three main factors that influence this syndrome: metabolic dysregulation, creating a negative energy balance; catabolism; and neuro-hormonal dysregulation. It is beyond the scope of this review to discuss these in-depth, but previous literature has detailed the pathophysiology of cancer cachexia [1, 44]. As outlined by Fearon et al. [1], cancer cachexia is a continuum which has three key stages, namely precachexia, cachexia, and refractory cachexia (Fig. 1).

The hallmark of weight loss associated with cancer cachexia is that there is a preferential loss of lean muscle mass and that the weight loss cannot be reversed with conventional feeding alone [43]. Thus, and in accordance with the consensus definition of cancer cachexia [1], merely assessing weight loss does not reflect the multidimensional syndrome of cancer cachexia. Furthermore, BMI has previously been used to assess for cachexia if weight loss is not known [45]. However, caution should be exercised in relation to this given the rise in prevalence of overweight and obese adults and children, which rose by 27.5% and 47.1% respectively between 1980 and 2013 [46].

Research focusing on gaining insight and understanding into the metabolic anomalies associated with cancer cachexia has proliferated in the last two decades. In parallel to this, clinical research efforts have focused on the potential management of cachexia aimed at reversing or ameliorating the associated weight loss. Most recently, the international Pre-MENAC and MENAC trials [43, 47], using a multimodal approach to cancer cachexia management, are reflective of the multifactorial syndrome itself [48]. However, preventative and therapeutic treatment options thus far have been shown to be largely ineffective and currently there is no notable pharmacological agent that successfully halts and reverses the weight loss associated with cancer cachexia. Furthermore, the optimal treatment for cachexia in patients with cancer has yet to be determined and currently there is no licensed treatment for cancer cachexia [43]. In practice today, despite much time and investment into clinical research, standardized local guidelines for the assessment, classification, and treatment of cachexia have still not been developed [49]. This only serves to compound the relevance of cachexia in negatively affecting both morbidity and mortality. Globally, cachexia is directly responsible for 20% of all cancer deaths, equating to more than 7.4 million deaths worldwide annually [50, 51].

## Cancer-Associated Weight Loss: Impact on Patients and Their Families

Cancer cachexia is associated with reduced functionality and adversely impacts on activities of daily living [52], unfavorable psychosocial impact [53], reduced tolerance to anticancer therapy [54], and reduced survival [55]. Furthermore, it impacts not only patients but also their family members [56]. In particular, the lack of understanding about the role of food in cancer cachexia management can lead to family conflicts and deleteriously affect family dynamics [57]. To date, the research which has explored the lived experience and impact of cachexia has focused on adult patients. While previous cancer studies with AYAs have highlighted cancer cachexia in relation to symptoms experienced [4, 58], no study has explored the impact of this syndrome in-depth with AYAs. Due to the developmental stage of AYAs, the holistic implications are likely to be far reaching and distinctive from adult patients. Most notably, the psychosocial challenges of this stage of maturity [4] coupled with the biopsychosocial ramifications of cancer cachexia [57] will make the impact unique from older age groups most commonly studied.

In relation to nutritional problems in cancer, previous descriptive research conducted with children and their parents (*n* = 69) has highlighted that parents as well as children need comprehensive and regular information [59]. Indeed, information on both diet and nutrition have been reported as an unmet among AYA patients with cancer [12]. This is reflective of work that has previously been conducted with an adult cancer cachexia population, where the patient and also the patient’s family needs support to cope with and provide care with nutritional issues [60]. The role of family may be all the more important for AYAs, as they may still be dependent on their families for food purchase and preparation and may experience “pressure to eat” from both family at home and through peer pressure from their social circle [61].

Previous research conducted in relation to communication about weight with adolescents outlines the sensitivities that need to be considered when engaging in communication about weight with this cohort. In particular, the need to avoid communication which induces negative emotional reactions is outlined [62]. This is a key message as previous research conducted with an adult cachectic population outlined negative labelling that family used and which inadvertently caused hurt or sadness such as “the look of someone from Belsen” [63]. Such terminology may be particularly damaging for AYAs with cachexia, particularity in relation to self-image and sexuality due to their stage of maturity. Thus, when providing additional information in relation to cancer cachexia to AYAs, both health care professionals and parents should be educated to identify and desist from using such negative terminology and assist in promoting a supportive dialogue in relation to the role of food and cancer-associated weight loss.

One of the defining characteristics of cachexia is weight loss that manifests as preferential loss of lean muscle mass, which is obvious visually. Given that AYAs are particularly concerned with how others see them and they often do not want to be seen as someone who is “sick,” addressing the visuality of cachexia is a key area of concern for future care consideration to support AYAs adjustment of body changes. The mode of delivery of such future care also needs to be addressed as many AYAs turn to the internet for both information and for support from peers [64]. Providing age-appropriate information and a forum for discussion in relation to cachexia is a necessity alongside age-appropriate person-centered health care [65] ensuring the values, goals, and preferences unique to this population are understood.

Cancer-associated weight loss is known to resonate with patients and family members and indeed health care professionals in terms of mortality [57, 66]. Progressive cachexia is indicative of a poorer prognosis and shorter survival time [67]. Within an adult cancer population, addressing cachexia may be avoided by health care professionals due to concern of it leading into an end of life conversation [68]. In relation to AYAs (with and without chronic illness), research has demonstrated that they are willing to discuss end of life decision-making so it is vital that it is addressed in clinical practice to provide quality end of life care [69]. The timeliness of such conversation is particularly relevant as previous AYAs with advanced cancer outlined that discussions about end of life were close to death and this negatively impacted on their ability to psychologically prepare for death [70]. Advance care planning is an essential part of care and this should encompass cachexia and its management, if this is relevant. The uniqueness of the AYAs population pertains to this aspect of care and end of life care preferences as these patients may differ from those of the adult population. Additional research is warranted to explore this further and develop age relevant resources to assist this process.

## Conclusion and Future Research Direction

There is a current dearth of information in relation to cancer-associated weight loss and AYAs. While previous work has been conducted in relation to cancer-associated weight loss in an adult population, this may not highlight the nuances in the AYAs population. Reflective of this, the management strategies’ needs for AYAs with cancer-associated weight loss are likely to be very different from an adult population. To help empower and optimize quality of life for AYAs with cancer-associated weight loss, person-centered care which recognizes and responds to the needs of AYAs with cancer-associated weight loss needs to be developed. Such care must be evidence based. To achieve this, it is essential to determine AYAs experience of cancer-associated weight loss and in particular, the role of their family and peers coupled with their age-related development (including bio-psycho-social-sexual domains) are key areas that require additional research. In planning future work in this area, a consensus definition of AYAs is needed to allow comparison across work and prevent unnecessary duplication of efforts. Given the social impact already known in relation to cancer cachexia and its impact on family dynamics (58; 54) and the psychosocial developmental stage that AYAs are at, it would be prudent to assess social functioning, family dynamics in AYAs with cancer-associated weight loss.
